# Nitric Oxide–cGMP Pathway Modulation in an Experimental Model of Hypoxic Pulmonary Hypertension

**DOI:** 10.1177/10742484211014162

**Published:** 2021-05-08

**Authors:** Melanie Reinero, Maurice Beghetti, Piergiorgio Tozzi, Ludwig K. von Segesser, Michele Samaja, Giuseppina Milano

**Affiliations:** 1 Department Cœur-Vaisseaux, Cardiac Surgery Center, University Hospital of Lausanne, Lausanne, Switzerland; 2 Unité de Cardiologie Pédiatrique, 30538 University Hospital of Geneva and Centre Universitaire Romand de Cardiologie et Chirurgie Cardiaque Pédiatrique University of Geneva and Lausanne, Switzerland; 3 Department of Surgery and Anesthesiology, Cardio-Vascular Research, Lausanne, Switzerland; 4 Department of Health Science, 9304 University of Milano, Milan, Italy

**Keywords:** l-arginine, NO-donors, PDE5 inhibitors, NO-cGMP pathway, hypoxia, pulmonary hypertension

## Abstract

Manipulation of nitric oxide (NO) may enable control of progression and treatment of pulmonary hypertension (PH). Several approaches may modulate the NO-cGMP pathway in vivo. Here, we investigate the effectiveness of 3 modulatory sites: (i) the amount of l-arginine; (ii) the size of plasma NO stores that stimulate soluble guanylate cyclase; (iii) the conversion of cGMP into inactive 5′-GMP, with respect to hypoxia, to test the effectiveness of the treatments with respect to hypoxia-induced PH. Male rats (n = 80; 10/group) maintained in normoxic (21% O_2_) or hypoxic chambers (10% O_2_) for 14 days were subdivided in 4 sub-groups: placebo, l-arginine (20 mg/ml), the NO donor molsidomine (15 mg/kg in drinking water), and phoshodiesterase-5 inhibitor sildenafil (1.4 mg/kg in 0.3 ml saline, i.p.). Hypoxia depressed homeostasis and increased erythropoiesis, heart and right ventricle hypertrophy, myocardial fibrosis and apoptosis inducing pulmonary remodeling. Stimulating anyone of the 3 mechanisms that enhance the NO-cGMP pathway helped rescuing the functional and morphological changes in the cardiopulmonary system leading to improvement, sometimes normalization, of the pressures. None of the treatments affected the observed parameters in normoxia. Thus, the 3 modulatory sites are essentially similar in enhancing the NO-cGMP pathway, thereby attenuating the hypoxia-related effects that lead to pulmonary hypertension.

## Introduction

**Figure 1.**
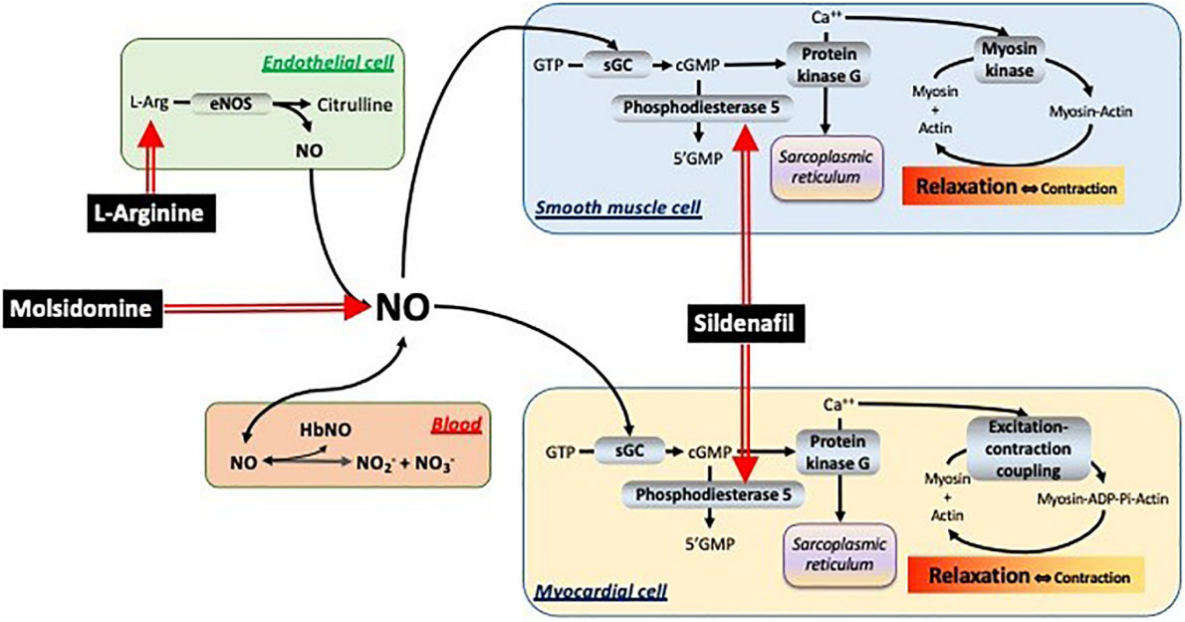
Simplified scheme of the mechanisms investigated in this study. Four distinct compartments are examined: the endothelial cells (green), the blood (red), the smooth muscle cell (light blue), and the myocardial cell (yellow). The modulators considered here (in black boxes) are l-arginine, which acts in endothelial cells, molsidomine, which acts on the NO stores in blood, and sildenafil, which inhibits phosphodiesterase-5 in smooth muscle and myocardial cells.

Pharmacological modulation of nitric oxide (NO)^1^ is well-known to be an efficient way to regulate the progression of several cardiopulmonary diseases and to represent a potential therapeutic option. Many studies suggest that NO bioavailability is indeed reduced in patients and in rodent models of pulmonary vascular diseases, and that enhancement of the NO-cyclic guanosine monophosphate (cGMP) pathway (Figure 1) may represent an effective strategy for treatment.^1-4^ NO is synthesized in endothelial cells from l-arginine (L-Arg) by endothelial NO synthase (eNOS).^5^ After its release in the circulation, it establishes an equilibrium with plasma nitrates and nitrites (NOx) and, to a minor extent, with circulating hemoglobin (Hb) to form nitrosylated Hb or nitrosothiolated Hb.^6^ Part of NO enters the smooth muscle cell, where it activates soluble guanylate cyclase (sGC) generating cGMP, which in turns activates protein kinase G that sequesters Ca^++^ thereby favoring smooth muscle relaxation.^7^ Although controversial,^8^ the same mechanism in the myocytes might contribute to decrease muscle contractility thereby acting as a negative inotropic factor. The cellular level of cGMP is kept under the control of the activity of phosphodiesterase 5 (PDE5), which converts active cGMP in inactive 5′-GMP.

The mentioned chain of events may be modulated at 3 levels at least, by altering: (i) the amount of L-Arg entering the chain; (ii) the amount of NO in the plasma that can stimulate sGC; or (iii) the amount of cGMP that is inactivated to 5′-GMP by PDE5. Several drugs have been proposed to manipulate the NO-cGMP pathway activity through 3 broad approaches: i) by manipulating the level of L-Arg, ii) by increasing the amount of plasma NO, for example through the use of NO donors, and iii) by modulating the activity of PDE5, for example through the use of PDE5 inhibitors. The NO-cGMP pathway has become a therapeutic target and several treatments, as inhaled NO, administration of PDE5 inhibitors and soluble guanylates cyclase modulators, have been proposed to treat several cardiopulmonary diseases, such as pulmonary arterial hypertension (PAH, group 1 of PH^9^) and neonatal respiratory diseases associated with PH.^10^ Although any one of the mentioned actions to exploit the NO-cGMP pathway-based therapies could be considered effective, in the clinical practice this does not occur, and a recent Cochrane survey shows that there is currently insufficient evidence to recommend NO donors, L-Arg or eNOS inhibitors in acute stroke, and only one drug, glyceryl trinitrate is able to give measurable clinical endpoints.^11^ Although inhaled NO is approved to treat certain types of PH and PDE5 inhibitors are approved in the treatment of PAH, the overlapping of several side reactions that eventually masks the final outcome also plays a role in this disappointing result.

The aim of this study is to investigate separately the effectiveness of the 3 modulations mentioned above with respect to a single cardiopulmonary stress, e.g. hypoxia, in order to understand how a single modulation affects the final outcome. To this purpose, here we examine the effects of manipulation of the NO pathway with regard to the chronic hypoxic in cardiopulmonary system. It is widely appreciated that NO production is limited by insufficient supply of L-Arg. When administered in a variety of cardiovascular diseases, L-Arg, NO donors and PDE5 inhibition appear to reduce PH progression in hypoxic animals and chronic heart failure patients. Yet, studies that compare these strategies using the same methods, approaches, procedures and kinds of animals with the same PH severity have not yet been performed. Therefore, this study aims at supporting the paradigm that pulmonary hemodynamics and gas exchange efficiency may benefit from NO-based approaches in patients with PH.

## Materials and Methods

### Chronic Hypoxia and Experimental Groups

Adult male Sprague-Dawley rats (n = 80; 10/group; 200-250 g; Charles River, France) were randomly assigned to 1 of 4 treatments: Control (no treatment), L-Arg (20 mg/ml in drinking water), the NO-donor molsidomine (Mols, 0.1875 mg/ml in drinking water), and the PDE5 inhibitor sildenafil (Sild, 1.4 mg/kg in 0.3 ml saline, i.p.). In each group, rats were maintained either in normoxic (21% O_2_) or hypoxic chambers (10% O_2_) for 14 days.^12,13^ The chambers, built to accommodate 4 rats each, differed only for the gas mixture flowing through them. When the hypoxic chambers needed to be opened for cleaning and monitoring, they were equipped with a compensation chamber flowed with the same gas mixture of the hypoxic chamber, thereby avoiding any contact of the rat with room air and its reoxygenation. Rats were treated with the assigned drug since day 0 of normoxia or hypoxia exposure. At day 15, rats were first transferred anaerobically to the compensation chamber, then anesthetized i.p. with a cocktail containing 10 mg/kg xylazine, 100 mg/kg ketasol and 1500 IU heparin within the compensation chamber at 10% O_2_, thus avoiding reoxygenation before sacrifice.^14^ In each subgroup, 2 subsets of rats were used, one for hemodynamic and morphological analyses, and one for tissue assays.

Animal experiments were performed according to the Swiss Federal guidelines (Ethical Principles and Guidelines for Experiments on Animals). The protocol was approved by the local Institutional Animal Committee (Service Vétérinaire Cantonal, Lausanne, Switzerland). All efforts were made to minimize animal suffering during the experiments.

### Right Heart Catheterization

To examine PH development, we measured the RV systolic pressure (RVSP) and the RV contractility expressed as maximum rate of pressure development (dP/dt_max_).^12^ Briefly, a small traverse incision was made in the abdominal wall, the diaphragm was exposed and opened. A 24-gauge butterfly needle with tubing attached to a pressure catheter was inserted into the RV and pressure measurements were recorded with PowerLab (AD Instruments, Colorado Springs, CO).

### Blood Collection and Analysis

At the end of the hemodynamic study, a blood sample was collected in heparinized syringes from the abdominal vein to measure Hb concentration, hematocrit and red blood cell count (Abbott Cell-dyn 3500 R System, Baar, Switzerland). The remaining sample was centrifuged for 15 min at 3000g. Plasma was stored at −80°C. For the measurements of NOx, we used the Nitric Oxide Colorimetric assay kit (Nitrate/Nitrite colorimetric Assay Kit, Cayman Chemical, No. 780001) following the manufacturer’s protocol.

### Heart Morphology

The anesthetized animals were sacrificed by exsanguination, heart and lung were washed with PBS at 4°C, harvested and processed for analysis. The RV was separated from the left ventricle (LV) and septum (S), and all parts were weighed separately. The degree of RV hypertrophy was calculated as the RV/LV+S ratio.

The degree of cardiac fibrosis was determined by Sirius red staining. Briefly, 10 µm-thick paraffin-embedded tissue sections were deparaffinized, rehydrated and stained in iron hematoxylin for 10 min, followed by rinsing in water for 10 min. The slides were then stained in 0.1% (w/v) Sirius Red F3B (Sigma-Aldrich, Buchs SG, Switzerland) in saturated aqueous picric acid (Sigma-Aldrich, Buchs SG, Switzerland) for 1 h. Sections were washed twice in 0.5% v/v acetic acid, dehydrated rapidly 3 times in 100% alcohol, cleared in xylene and mounted using Merckoglas medium (Merck, Germany). Each section was photographed under a light microscope (Nikon Instruments Inc., Melville, NY, USA) at 10x magnification. At least 10 fields were randomly selected for each rat, each of which contains at least 6 vessels. Images were analyzed using Image software. The percentage collagen was calculated as fibrosis area/total area of the tissue section.

### Pulmonary Vascular Remodeling

To determine the degree of muscularization in pulmonary arterioles, we used the technique described in Nydegger et al,^12^ which includes lung inflation with formalin, embedding in paraffin and sectioning (8-µm thickness). Samples were then blocked with goat serum and incubated with an antibody against smooth muscle α-actin (α-SMA 1:250, clone 1A4, Sigma-Aldrich), followed by incubation with goat anti-mouse IgG secondary antibody (1/500, DAKO). After developing with 3,3′-diaminobenzidine and counter staining with hematoxylin and eosin, transversal cut arterioles were counted (image analysis system Nikon eclipse 80i camera with NIH image software, Nikon Instruments Inc., Melville, NY, USA). Pulmonary arterial thickening was measured by calculating the percentual pulmonary artery thickness as described in the literature.^12^ We analyzed 10 vessels for each rat (n = 6/group) by double-blind morphological analysis.

### cGMP and Caspase-3 Activity

We measured cGMP in frozen tissue that was homogenized at 4°C with 0.1 mol·l^−1^ HCl (10% wet wt/vol) and centrifuged (2000 rpm for 10 min at 4°C). The measurement of cGMP was made by an immunoassay kit (Assay designs, Inc., MI). Caspase-3 activity was tested by the Caspase-3/CPP32 colorimetric assay kit (MBL). NOx was determined by the Nitric Oxide Colorimetric assay kit (Nitrate/Nitrite colorimetric Assay Kit, Cayman Chemical, No. 780001) following the manufacturer’s protocol.

### Statistics

Data are expressed as box and whiskers plot following the Tukey method, with outliers plotted individually when present. To detect significant differences, we first ran ordinary 2-way ANOVA analysis. If significant, we proceeded to the multiple comparison tests. To test whether hypoxia has effect on the single treatments, we used the Sidak’s multiple comparison test (significant comparisons are marked as # in the figures). To detect whether treatments had an effect with respect to controls within normoxia and hypoxia, we used the Dunnett’s test (significant comparisons are marked as § in the figures). Analyses were performed using GraphPad Prism version 8, San Diego, California USA. The significance level was set at *P* = 0.05.

## Results

None of the rats that entered this study (243.7 ± 1.4 g body weight, BW) died nor showed signs of discomfort. Unless otherwise stated, the treatments did not have remarkable effects in normoxia rats.

### Systemic Changes: Homeostasis, Erythropoiesis, and Plasma NOx

**Figure 2.**
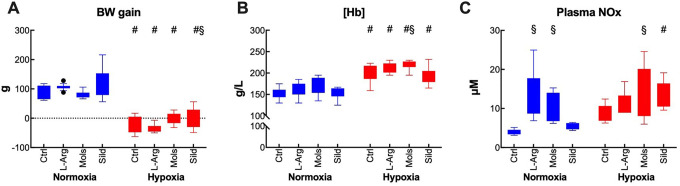
Some systemic effect of hypoxia (14 days at 10% O_2_) in rats treated with placebo (control), l-arginine (L-Arg), molsidomine (Mols), and sildenafil (Sild) for the same time duration. Panels A, B, and C report the effects on the body weight gain, hemoglobin concentration, and plasma nitrates and nitrites, respectively. Data are expressed as box and whiskers plots following the Tukey method, with outliers plotted individually when present. ^#^ *P* < 0.05: normoxia vs hypoxia within the same treatment (Sidak’s test); ^§^ *P* < 0.05: with respect to control within normoxia or hypoxia (Dunnett’s test).

Fourteen-day hypoxia depressed the BW gain and increased (Hb) concentration ([Hb]) (Figure 2A and B). Plasma NOx expresses the level of NO stores in blood (Figure 2C). As expected, both L-Arg and Mols increased plasma NOx in normoxia, as opposed to lack of effect of Sild, which affects the NO-cGMP pathway downstream.

The treatments in hypoxia did not substantially affect neither the BW gain nor [Hb], although Sild slightly improved the BW gain and Mols slightly increased [Hb]. L-Arg and Sild were unable to alter plasma NOx, but Mols further increased it by virtue of its NO-donor effect. Although Sild did not change plasma NOx with respect to untreated hypoxic rats, plasma NOx was higher than in normoxia.

### Myocardial Morphology

**Figure 3.**
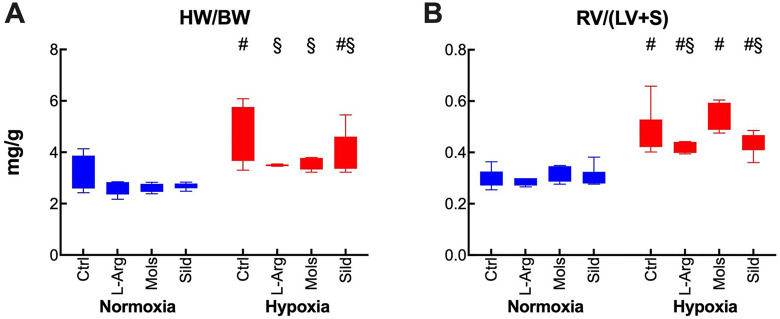
Effects of hypoxia (14 days at 10% O_2_) on myocardial morphology in rats treated with placebo (control), l-arginine (L-Arg), molsidomine (Mols), and sildenafil (Sild) for the same time duration. Panels A and B report the effects on the heart/body weight ratio and the right ventricle/(left ventricle + septum) ratio, respectively. Data are expressed as box and whiskers plots following the Tukey method, with outliers plotted individually when present. ^#^ *P* < 0.05: normoxia vs hypoxia within the same treatment (Sidak’s test); ^§^ *P* < 0.05: with respect to control within normoxia or hypoxia (Dunnett’s test).

Myocardial hypertrophy was evaluated through the heart weight/body weight (HW/BW). Hypoxia markedly increased the HW/BW ratio (Figure 3A). This increase was at least in part due to RV hypertrophy, which was evaluated through the RV/(LV+S) ratio (Figure 3B). The increase in both myocardial and RV hypertrophy was mitigated by all treatments, while Mols did not apparently affect the RV/(LV+S) ratio.

**Figure 4.**
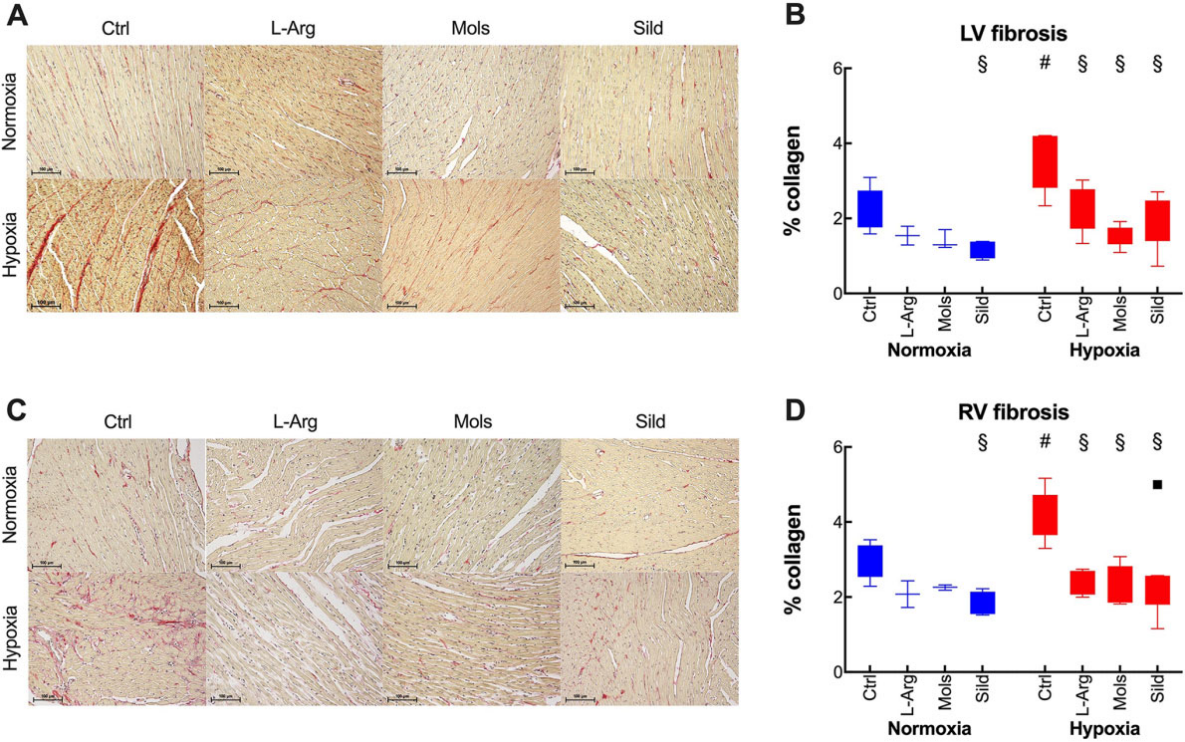
Effects of hypoxia (14 days at 10% O_2_) on myocardial fibrosis in rats treated with placebo (control), l-arginine (L-Arg), molsidomine (Mols), and sildenafil (Sild) for the same time duration. Panels A and B refer to the left and right ventricles, respectively. Panels A and C report representative pictures, with the bar representing 100 μm. Summary data in panels B and C are expressed as box and whiskers plots following the Tukey method, with outliers plotted individually when present. ^#^ *P* < 0.05: normoxia vs hypoxia within the same treatment (Sidak’s test); ^§^ *P* < 0.05: with respect to control within normoxia or hypoxia (Dunnett’s test).

Myocardial fibrosis was evaluated separately in the RV and LV by Sirius Red Staining (Figure 4). Hypoxia increased myocardial fibrosis in both the LV and RV. All treatments could normalize myocardial fibrosis.

### Biochemistry

**Figure 5.**
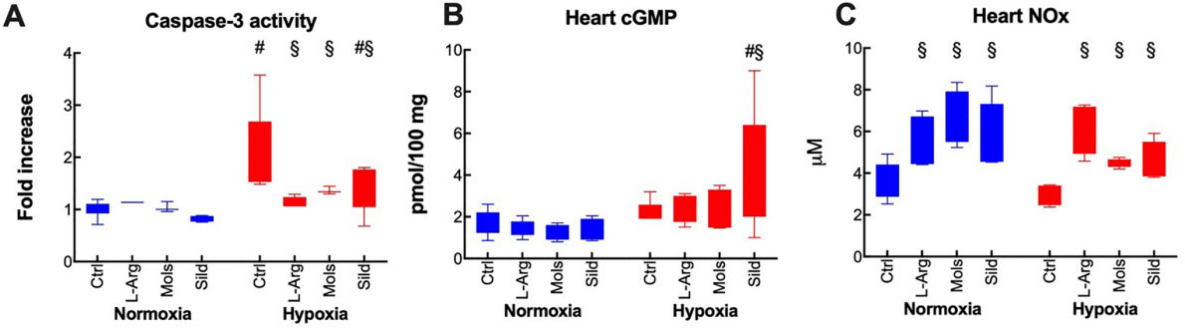
Effects of hypoxia (14 days at 10% O_2_) on myocardial biochemistry in rats treated with placebo (control), l-arginine (L-Arg), molsidomine (Mols), and sildenafil (Sild) for the same time duration. Panels A, B, and C report the effects on Caspase 3, myocardial cGMP content, and the myocardial NOx level, respectively. Data are expressed as box and whiskers plots following the Tukey method, with outliers plotted individually when present. ^#^ *P* < 0.05: normoxia vs hypoxia within the same treatment (Sidak’s test); ^§^ *P* < 0.05: with respect to control within normoxia or hypoxia (Dunnett’s test).

The degree of apoptosis was evaluated through the activation of Caspase-3, a hallmark of apoptosis in myocardial cells (Figure 5A). Hypoxia determined a marked increase in myocardial apoptosis. Myocardial cGMP, which is directly related to the modulation of PDE5 by its inhibitors, was not affected by hypoxia (Figure 5B). The level of myocardial NOx remained nearly unaffected by hypoxia, but all treatments elevated this level with respect to control groups (Figure 5C).

All treatments were able to normalize apoptosis in hypoxia-challenged hearts. By contrast, the myocardial cGMP level was increased only by Sild, as a result of its function as PDE5 inhibitor. All treatments increased the production of NO.

### Hemodynamics

**Figure 6.**
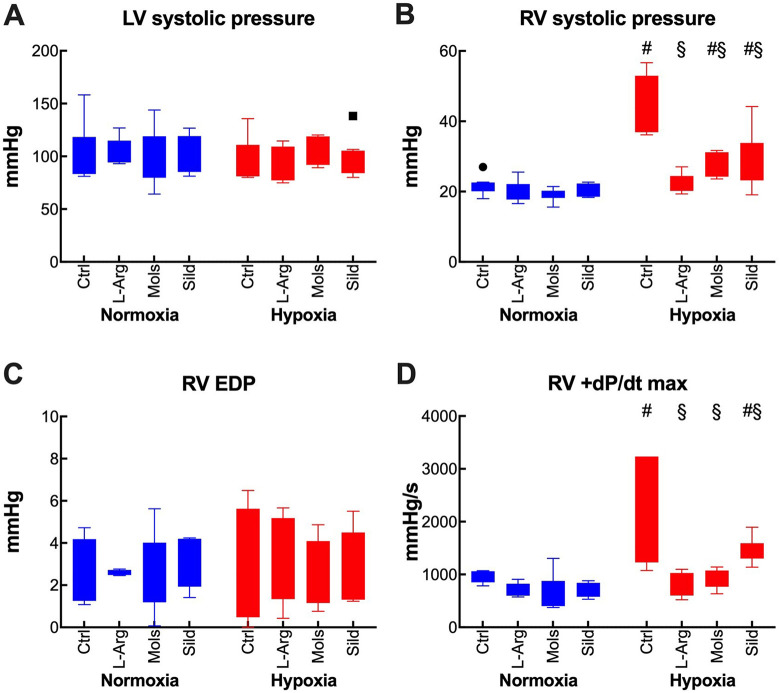
Effects of hypoxia (14 days at 10% O_2_) on hemodynamics and myocardial function in rats treated with placebo (control), l-arginine (L-Arg), molsidomine (Mols), and sildenafil (Sild) for the same time duration. Panels A, B, C, and D report the effects on the left ventricle systolic pressure, the right ventricle systolic pressure, the right ventricle end-diastolic pressure, and the maximal rate of pressure development in the right ventricle, respectively. Data are expressed as box and whiskers plots following the Tukey method, with outliers plotted individually when present. ^#^ *P* < 0.05 normoxia vs hypoxia within the same treatment (Sidak’s test); ^§^ *P* < 0.05 with respect to control within normoxia or hypoxia (Dunnett’s test).

Hemodynamics was assessed by left and right heart catheterization with measure of the end-diastolic (EDP) and systolic (SP) pressures, and of the rate of pressure development (dP/dt_max_). Hypoxia did not affect the left heart function (Figure 6A), but RV SP (Figure 6B) and RV dP/dt_max_ were markedly increased (Figure 6C), indicative of pulmonary pressure increase. RV EDP remained unaffected (Figure 6C). LV SP was not affected by none of the treatments. By contrast, all treatments decreased RV SP and RV +dP/dt_max_ (Figure 6D) without effects on RV EDP. All hemodynamics parameters were recorded in rats breathing the same gas mixture, hypoxic or normoxic, to which they were exposed during the preceding 14 days using a technology that fully prevents any risk of reoxygenation.^14,15^

### Pulmonary Remodeling

**Figure 7.**
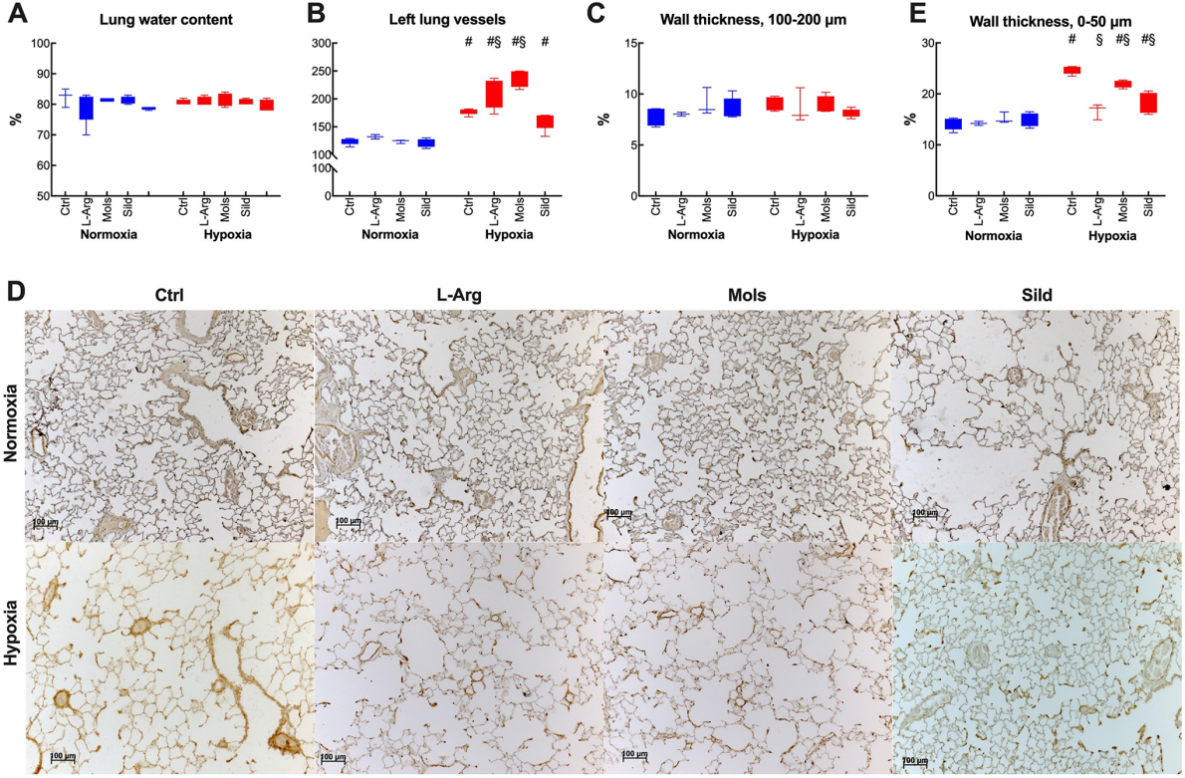
Effects of hypoxia (14 days at 10% O_2_) on lung morphology and remodeling in rats treated with placebo (control), l-arginine (L-Arg), molsidomine (Mols), and sildenafil (Sild) for the same time duration. Panels A, B, C, and E report the effects on the lung water content, the number of vessels in the left lung, the average wall thickness in large-caliber vessels, and the average wall thickness in small-caliber vessels, respectively. Panel D reports representative images from which the vessels numbers were derived. Data are expressed as box and whiskers plots following the Tukey method, with outliers plotted individually when present. ^#^ *P* < 0.05: normoxia vs hypoxia within the same treatment (Sidak’s test); ^§^ *P* < 0.05: with respect to control within normoxia or hypoxia (Dunnett’s test). The pictures report representative images of lung vessels in the 0-50 µm range.

The lung water content highlights the presence of pulmonary edema, which was not affected by hypoxia (Figure 7A). To evaluate pulmonary remodeling, we measured the number of vessels in lung sections stained with hematoxylin & eosin (Figure 7B), as well as the medial wall thickness in large (100-200 µm internal diameter, Figure 7D) and small (internal diameter 0-50 μm, Figure 7E) pulmonary vessels. The figure reports the number of vessels in the left lung only because this value was superimposable to that found in the right lung (not shown). Hypoxia markedly increased the number of vessels in the left lung as well as the medial wall thickness of small pulmonary arteries, while pulmonary vessels of larger caliber remained unaffected, confirming previously published data.^13^

The effect of the treatments was heterogenous. While the lung water content remained unchanged, the number of vessels in the left lung was unaffected by Sild, but L-Arg and Mols further increased it. The wall thickness in small vessels was almost normalized by all the treatments, as opposed to lack of effect in large vessels.

## Discussion

In this study, we investigate 3 of the broad mechanisms that enhance the NO-cGMP pathway activity in hearts and lungs challenged by chronic hypoxia. As expected, 14-day breathing a gas mixture containing 10% O_2_, equivalent to approximately 5000 m altitude, affects most of the considered parameters without being lethal. The degree of hypoxia can therefore be classified as moderate, but still potentially harmful. Therefore, the selected hypoxia condition results in a layout that includes depressed body homeostasis, excessive erythropoiesis, heart and RV hypertrophy, myocardial fibrosis, higher degree of apoptosis and as a stimulus for pulmonary vascular remodeling. These features lead to a phenotype very similar to PH in humans. As drug treatments started in coincidence with the onset of hypoxia, the observed mechanisms may reflect prophylactic rather than therapeutic interventions.

The stimulation of anyone of the 3 broad mechanisms that enhance the NO-cGMP pathway did not produce appreciable changes in normoxia, but tended to improve, and in some instance to normalize most of the parameters that were challenged by hypoxia. This highlights the validity of the experimental model with respect to the pathological situation of PH patients. Remarkably, monocrotaline-treated rats, a common rodent model of PH, develop symptoms similar to those elicited by hypoxia only, including pulmonary vessel remodeling, RV hypertrophy and apoptosis.^12,13,16^ However, hypoxia-induced PH occurs without use of compounds that were reviewed to be subjected to differences in pharmacokinetics, hepatic formation of pyrrole derivatives that finally lead to anorexia, apathy, inability to gain weight and tachypnea, all symptoms that may overlap with those led by hypoxia as dyspnea, weakness, diarrhea, and peripheral cyanosis.^17^ In addition to monocrotaline-treated rats, the Sugen/Hypoxia model also mimics PAH in group 1 by combining a single administration of Sugen 5416, a vascular endothelial growth factor receptor 2 (VEGFR2) antagonist, with 3-week hypoxia. This treatment causes severe PAH in rats, characterized by smooth muscle proliferation, vascular rarefaction and perivascular fibrosis that contribute to increase precapillary vascular resistance, RV afterload and hypertrophy even after return to normoxia for 5-10 weeks.^18,19^ Compared with rats exposed to chronic hypoxia alone, Su-Hx rats develop decompensated RV failure, as evident from elevated RV EDP, RV dilatation and decreased RV ejection fraction with maladaptative RV remodeling.^20^ A third PH model is constituted by the persistent PH model obtainable in lambs by surgical constriction of the fetal ductus arteriosus,^21,22^ which results in increased right-to-left extra-pulmonary shunting of deoxygenated blood and mimics with augmented vascular constriction and smooth muscle hypertrophy, which leads to persistently increased pulmonary vascular resistance and RV hypertrophy.

In this study, rats were exposed to hypoxia for 14 days, a time long enough to rise some adaptation to hypoxia or compensation to PH. Therefore, data taken at a single time point may be considered potentially unstable and not fully representative of hypoxia-induced PH. However, we previously showed^12^ that in this model most of the data reported here remain constant for 4 weeks. Despite ongoing hypoxia adaption as from the steadily increasing erythropoietic response, most parameters related to the compensation to PH appear relatively constant, including the RV/(LV+S) ratio, the number of lung vessels, the wall thickness of small vessels, RV fibrosis, and myocardial NOx. Remarkably, the effect of one of the interventions described here, e.g. PDE5 inhibition, elicits similar responses after 2- and 4-week hypoxia. Noteworthily, despite constant LV SP and RV SP, LV EDP and dP/dt_max_ were subjected to instability on opposite directions: tendency toward normalization for dP/dt_max_, and toward further deterioration for LV EDP.

PH patients, in particular group 3 due to hypoxia or lung disease, usually display low L-Arg levels compared to healthy subjects.^4,23^ Consequently, increasing the substrate for NO production (L-Arg supplementation) is thought to attenuate PH development in both monocrotaline-^24^ and hypoxia-challenged^25^ rats. In PH patients, L-Arg supplementation considerably reduces pulmonary vascular resistance.^26^ It must be noted that L-Arg supplementation can be replaced by other treatments or supplementation, as for example combining l-citrulline with tetrahydrobiopterin, which produced encouraging results in hypoxic pigs.^27^ Besides providing the substrate for NO formation in endothelial cells, L-Arg prevents eNOS uncoupling, a phenomenon whereby eNOS generates ROS rather than NO, thus serving as ROS scavenger, decreasing their formation and increasing NO release.^28^ In this study, L-Arg attenuated RV hypertrophy and muscularization of small pulmonary vessels secondary to the effect of NO in inhibiting vascular pulmonary artery smooth muscle cell proliferation and attenuating the morphological changes by improving cardiopulmonary remodeling. It should be noted that, besides providing substrate for eNOS, L-Arg also feeds the inducible form of NO synthase (iNOS), which, at odds with eNOS, generates considerable oxidative and nitrosative stress.^29^

NO donors were first reviewed in 1995 and classified as “a new class of drugs […with…] potential utility in the treatment of coronary and pulmonary arterial diseases.”^30^ Among several developed NO donor molecules, Mols, which releases NO non-enzymatically, was suggested for acute pre-ischemic treatment that improves cardiac ischemia tolerance in animals adapted to hypoxia.^31^ Several reports have shown that Mols reduces the pulmonary arterial pressure in rats with PH induced by monocrotaline injection or hypoxia^32^ and reduces both pulmonary vascular remodeling and ET-1 expression.^33^ In addition, Mols improves cardiac function, reduces neurological symptoms and enhances atherosclerotic plaque stability.^34^ In this study, we found that Mols acts similarly to L-Arg, but, in addition, it provides a further stimulus to erythropoiesis. This is in agreement with the observation that the NO-cGMP pathway upregulates erythropoiesis at the level of gene transcription, thereby providing a novel target to stimulate erythropoiesis in vivo.^35^

It was reported that inhibition of PDE5 by Sild alleviates symptoms in several cardiopulmonary diseases including PH^36^ and rescues hypoxia-induced RV hypertrophy.^37^ This phenotype is the outcome of the increase of cGMP secondary to upregulated eNOS phosphorylation,^38-43^ which translates into mitigated apoptosis. Sild does not only contribute to vasodilation, but also favors the recruitment of bone marrow-derived c-kit+ cells, that improves pulmonary hemodynamic^16^ through a mechanism that persists over time, thus independent from hypoxia adaptation.^12^ Sild improves pulmonary remodeling and RV function in hypoxia by increasing NO signaling.^12,13^

In this study, we did not attempt dose/response assays to detect the drugs optimal concentration, but rather we approximated the dose normally used in clinical contexts. Assuming a water intake of 20 ml/day in hypoxic rats,^14^ the selected dose of L-Arg (1.6 g/day/kg for 2 weeks) compares with that associated with improved resting systolic blood pressure and quality of life in PH patients with microvascular angina (0.1 g/kg/day for 4 weeks).^44^ Similar doses were also effective to improve hemodynamics and exercise capacity in patients with precapillary PH (up to 0.15 g/kg)^45^ as well as to ameliorate functional and quality of life outcomes (0.1 g/kg/day for 3 months).^46^ Likewise, the selected dose of Mols (15 mg/kg/day) compares with that reported in the MEDCOR clinical trial demonstrating beneficial effects on the myeloperoxidase activity/antigen ratio in angina patients undergoing percutaneous coronary intervention (16 mg/day for 1 year)^47^ and is identical to that reported to be effective to reduce PH in hypoxic rats.^48^ As for Sild, the typical per os dose used for PAH treatment is usually higher (25-100 mg/kg twice-daily per os) than that used in the present study. However, for consistency with previous studies, we opted for i.p. delivery of Sild in a single daily dose within the range that saturates protection in humans.^49^ Despite the plasma half-life of 4-6 h,^50^ the selected dose was observed to elevate myocardial cGMP in a rodent model of hypoxia,^37^ indicative of efficient inhibition of PDE5 activity. Other /agents such as the stimulator of sGC riociguat might soon be competitive.^51^ This consideration is further supported by the observation that deactivating by oxidation the α subunit of protein kinase G does not change the benefits of the stimulation of sGC, thereby highlighting the combination therapy with PDE5 inhibitors and sGC stimulators.^52^ Here we show that the effect of Sild is virtually indistinguishable from those of L-Arg and Mols, with a slight improvement of body homeostasis. As expected, Sild did not affect the parameters upstream PDE5 inhibition, e.g. neither plasma nor myocardial NOx.^53^

Table 1 summarizes the most important findings in this study. The effect of the 3 mechanisms that enhance the NO-cGMP pathway, thus, followed the effects expected by the actions exerted by NO, which include reduction of heart and RV hypertrophy, apoptosis and pulmonary remodeling. Remarkably, the 3 mechanisms have an anisotropic effect in reducing the RV pressure and dP/dt_max_. In facts, in addition to the well-known effect of vasodilatation, an additional effect of NO on skeletal muscles fibers has been proposed, i.e. through nitrosation/nitrosylation of target proteins, which leads to depressed isometric force, shortened velocity of contraction, with the outcome of “braking” muscle contraction^54^ and lowering force output by altering the excitation-contraction coupling.^55^ By contrast, other Authors documented lack of inotropic effect of NO in rat myocardium.^56^

**Table 1.** Summary of the Observed Findings.^a^

		Hypoxia vs normoxia	Within hypoxia vs control		
			l-Arginine	Molsidomine	Sildenafil
Systemic changes	Body homeostasis	↓	=	=	↑
	Erythropoiesis	↑	=	↑	=
	Plasma nitrates + nitrites	=	=	↑	=
Myocardial morphology	Cardiac hypertrophy	↑	↓↓	↓↓	↓
	RV hypertrophy	↑	↓	=	↓
	LV fibrosis	↑	↓↓	↓↓	↓↓ ^N^
	RV fibrosis	↑	↓↓	↓↓	↓↓
Heart biochemistry	Caspase-3 activation	↑	↓↓	↓↓	↓
	Heart cGMP	=	=	=	↑
	Myocardial NOx	=	↑↑	↑↑	↑↑
Myocardial function	LV systolic pressure	=	=	=	=
	RV systolic pressure	↑	↓↓	↓	↓
	RV end-diastolic pressure	=	=	=	=
	RV +dP/dt_max_	↑	↓↓	↓↓	↓
Pulmonary remodeling	Lung hypertrophy	↑	=	=	=
	Pulmonary remodeling, small vessels (<50 μm)	↑	↓↓	↓	↓
	Pulmonary remodeling, large vessels (100-200 μm)	=	=	=	=

^a^ ↑, increase; ↓, decrease; =, no change. For within hypoxia: ↓↓ and ↑↑ mean complete normalization (decrease or decrease, respectively) with respect to normoxia control. ^N^ denotes that the effect was observed also in normoxia.

### Limitations of the Study

In this study, no attempt was made to compare directly the effectiveness of the 3 strategies to improve the NO-cGMP pathway. This goal should be preceded by complex pharmacokinetic evaluations as well as dose/response preliminary studies. Likewise, a large number of drugs and approaches were not examined in this study, but we focused in a representative drug for every site of modulation. As far as NO donors are considered, alternative drugs/treatments include inhaled NO therapy,^57^ administration of inorganic nitrite and nitrate,^58^ such as sodium nitrite,^59^ as well as diethylamine NONOate diethylammonium, sodium nitroprusside and the sGC stimulator riociguat.^60^ As for PDE5 inhibitors, several other drugs besides Sild have been proposed, each with different pharmacokinetics, efficiency and persistence in the circulation, but with essentially similar mechanisms of action and outcomes.^61^ Finally, in this study we did not assess any combination of these approaches in the search of synergistic effects.

## Conclusion

Enhancing the NO-cGMP pathway in any form attenuates the hypoxia-related PH syndrome. Remarkably, the tested treatments appeared to be effective in hypoxia at doses that were ineffective in normoxia. Thus, the 3 strategies to enhance the NO-cGMP pathway in vivo appear equivalent in terms of efficacy and molecular mechanisms of action. The choice of the best treatment, therefore, should not be based on the mechanisms of action but rather on clinical relevance, such as tolerability, side effects and persistence in the circulation.

## References

[1] Lai YC Potoka KC Champion HC Mora AL Gladwin MT . Pulmonary arterial hypertension: the clinical syndrome. Circ Res. 2014;115(1):115–130. 24951762 10.1161/CIRCRESAHA.115.301146 PMC4096686

[bibr2-10742484211014162] Coggins MP Bloch KD . Nitric oxide in the pulmonary vasculature. Arterioscler Thromb Vasc Biol. 2007;27(9):1877–1885. 17541026 10.1161/ATVBAHA.107.142943

[bibr3-10742484211014162] Budhiraja R Tuder RM Hassoun PM . Endothelial dysfunction in pulmonary hypertension. Circulation. 2004;109(2):159–165. 14734504 10.1161/01.CIR.0000102381.57477.50

[4] Klinger JR Kadowitz PJ . The nitric oxide pathway in pulmonary vascular disease. Am J Cardiol. 2017;120(8S):S71–SS9. 29025573 10.1016/j.amjcard.2017.06.012

[5] Palmer R Ashton D Moncada S . Vascular endothelial cells synthesize nitric oxide from L-arginine. Nature. 1988;333:664–666. 3131684 10.1038/333664a0

[6] Gladwin M Ognibene F Pannell L , et al. Relative role of heme nitrosylation and beta-cysteine 93 nitrosation in the transport and metabolism of nitric oxide by hemoglobin in the human circulation. Proc Natl Acad Sci USA. 2000;97:9943–9948. 10954746 10.1073/pnas.180155397 PMC27634

[7] Murad F Leitman DC Bennett BM Molina C Waldman SA . Regulation of guanylate cyclase by atrial natriuretic factor and the role of cyclic GMP in vasodilation. Am J Med Sci. 1987;294(3):139–143. 2889359 10.1097/00000441-198709000-00003

[8] Sarkar D Vallance P Harding SE . Nitric oxide: not just a negative inotrope. Eur J Heart Fail. 2001;3(5):527–534. 11595600 10.1016/s1388-9842(01)00163-5

[9] Kuroda K Akagi S Nakamura K Sarashina T Ejiri K Ito H . Successful transition from phosphodiesterase-5 inhibitors to riociguat without a washout period in patients with pulmonary arterial hypertension and chronic thromboembolic pulmonary hypertension: a pilot cohort study. Heart Lung Circ. 2020;29(3):331–336. 30773322 10.1016/j.hlc.2019.01.013

[10] Dillard J Perez M Chen B . Therapies that enhance pulmonary vascular NO-signaling in the neonate. Nitric Oxide. 2020;95:45–54. 31870967 10.1016/j.niox.2019.12.003 PMC6980762

[11] Bath PM Krishnan K Appleton JP . Nitric oxide donors (nitrates), L-arginine, or nitric oxide synthase inhibitors for acute stroke. Cochrane Database Syst Rev. 2017;4:Cd000398. 28429459 10.1002/14651858.CD000398.pub2 PMC6478181

[12] Nydegger C Corno AF von Segesser LK Beghetti M Samaja M Milano G . Effects of PDE-5 inhibition on the cardiopulmonary system after 2 or 4 weeks of chronic hypoxia. Cardiovasc Drugs Ther. 2019;33(4):407–414. 31264002 10.1007/s10557-019-06887-9 PMC6689028

[13] Nydegger C Martinelli C Di Marco F , et al. Phosphodiesterase-5 inhibition alleviates pulmonary hypertension and basal lamina thickening in rats challenged by chronic hypoxia. Front Physiol. 2018;9:289. 29636700 10.3389/fphys.2018.00289 PMC5880920

[14] Milano G Corno AF Lippa S Von Segesser LK Samaja M . Chronic and intermittent hypoxia induce different degrees of myocardial tolerance to hypoxia-induced dysfunction. Exp Biol Med (Maywood). 2002;227(6):389–397. 12037128 10.1177/153537020222700604

[15] Milano G Abruzzo PM Bolotta A , et al. Impact of the phosphatidylinositide 3-kinase signaling pathway on the cardioprotection induced by intermittent hypoxia. PLoS One. 2013;8(10):e76659. 24124584 10.1371/journal.pone.0076659 PMC3790757

[16] Favre S Gambini E Nigro P , et al. Sildenafil attenuates hypoxic pulmonary remodelling by inhibiting bone marrow progenitor cells. J Cell Mol Med. 2017;21(5):871–880. 27860185 10.1111/jcmm.13026 PMC5387166

[17] Bello-Klein A Mancardi D Araujo AS Schenkel PC Turck P de Lima Seolin BG . Role of redox homeostasis and inflammation in the pathogenesis of pulmonary arterial hypertension. Curr Med Chem. 2018;25(11):1340–1351. 29278203 10.2174/0929867325666171226114838

[18] Taraseviciene-Stewart L Kasahara Y Alger L , et al. Inhibition of the VEGF receptor 2 combined with chronic hypoxia causes cell death-dependent pulmonary endothelial cell proliferation and severe pulmonary hypertension. FASEB J. 2001;15(2):427–438. 11156958 10.1096/fj.00-0343com

[19] Abe K Toba M Alzoubi A , et al. Formation of plexiform lesions in experimental severe pulmonary arterial hypertension. Circulation. 2010;121(25):2747–2754. 20547927 10.1161/CIRCULATIONAHA.109.927681

[20] Legchenko E Chouvarine P Borchert P , et al. PPARγ agonist pioglitazone reverses pulmonary hypertension and prevents right heart failure via fatty acid oxidation. Sci Transl Med. 2018;10(438):eaao0303. 29695452 10.1126/scitranslmed.aao0303

[21] Teng RJ Du J Welak S , et al. Cross talk between NADPH oxidase and autophagy in pulmonary artery endothelial cells with intrauterine persistent pulmonary hypertension. Am J Physiol Lung Cell Mol Physiol. 2012;302(7):L651–L663. 22245997 10.1152/ajplung.00177.2011 PMC3330765

[22] Ke X Johnson H Jing X , et al. Persistent pulmonary hypertension alters the epigenetic characteristics of endothelial nitric oxide synthase gene in pulmonary artery endothelial cells in a fetal lamb model. Physiol Genomics. 2018;50(10):828–836. 30004838 10.1152/physiolgenomics.00047.2018 PMC6230868

[23] Sandqvist A Schneede J Kylhammar D , et al. Plasma L-arginine levels distinguish pulmonary arterial hypertension from left ventricular systolic dysfunction. Heart Vessels. 2018;33(3):255–263. 28975394 10.1007/s00380-017-1055-7 PMC5847178

[24] Ou ZJ Wei W Huang DD , et al. L-arginine restores endothelial nitric oxide synthase-coupled activity and attenuates monocrotaline-induced pulmonary artery hypertension in rats. Am J Physiol Endocrinol Metab. 2010;298(6):E1131–E1139. 20215577 10.1152/ajpendo.00107.2010

[25] Howell K Costello CM Sands M Dooley I McLoughlin P . L-Arginine promotes angiogenesis in the chronically hypoxic lung: a novel mechanism ameliorating pulmonary hypertension. Am J Physiol Lung Cell Mol Physiol. 2009;296(6):L1042–L1050. 19346433 10.1152/ajplung.90327.2008

[26] Morris CR Morris SM Jr Hagar W , et al. Arginine therapy: a new treatment for pulmonary hypertension in sickle cell disease? Am J Respir Crit Care Med. 2003;168(1):63–69. 12626350 10.1164/rccm.200208-967OC

[27] Dikalova A Aschner JL Kaplowitz MR Cunningham G Summar M Fike CD . Combined L-citrulline and tetrahydrobiopterin therapy improves NO signaling and ameliorates chronic hypoxia-induced pulmonary hypertension in newborn pigs. Am J Physiol Lung Cell Mol Physiol. 2020;318(4):L762–L772. 32073878 10.1152/ajplung.00280.2019 PMC7191483

[28] Gielis JF Lin JY Wingler K Van Schil PE Schmidt HH Moens AL . Pathogenetic role of eNOS uncoupling in cardiopulmonary disorders. Free Radic Biol Med. 2011;50(7):765–776. 21172428 10.1016/j.freeradbiomed.2010.12.018

[29] Maxwell AJ . Mechanisms of dysfunction of the nitric oxide pathway in vascular diseases. Nitric Oxide. 2002;6(2):101–124. 11890735 10.1006/niox.2001.0394

[30] Loskove JA Frishman WH . Nitric oxide donors in the treatment of cardiovascular and pulmonary diseases. Am Heart J. 1995;129(3):604–613. 7872193 10.1016/0002-8703(95)90291-0

[31] Alanova P Kolar F Ostadal B Neckar J . Role of NO/cGMP signaling pathway in cardiac ischemic tolerance of chronically hypoxic rats. Physiol Res. 2015;64(5):783–787. 25804095 10.33549/physiolres.932939

[32] Mathew R Gloster ES Sundararajan T Thompson CI Zeballos GA Gewitz MH . Role of inhibition of nitric oxide production in monocrotaline-induced pulmonary hypertension. J Appl Physiol (1985). 1997;82(5):1493–1498. 9134898 10.1152/jappl.1997.82.5.1493

[33] Blumberg FC Wolf K Sandner P Lorenz C Riegger GA Pfeifer M . The NO donor molsidomine reduces endothelin-1 gene expression in chronic hypoxic rat lungs. Am J Physiol Lung Cell Mol Physiol. 2001;280(2):L258–L263. 11159004 10.1152/ajplung.2001.280.2.L258

[34] Roth L Van der Donckt C Emini Veseli B , et al. Nitric oxide donor molsidomine favors features of atherosclerotic plaque stability and reduces myocardial infarction in mice. Vascul Pharmacol. 2019;118-119:106561. 31085310 10.1016/j.vph.2019.05.001

[35] Ikuta T Sellak H Odo N Adekile AD Gaensler KM . Nitric oxide-cGMP signaling stimulates erythropoiesis through multiple lineage-specific transcription factors: clinical implications and a novel target for erythropoiesis. PLoS One. 2016;11(1):e0144561. 26727002 10.1371/journal.pone.0144561 PMC4699757

[36] Guazzi M Samaja M . The role of PDE5-inhibitors in cardiopulmonary disorders: from basic evidence to clinical development. Current Med Chem. 2007;14(20):1893–1910. 10.2174/092986707781389619 17691956

[37] Milano G Bianciardi P Rochemont V , et al. Phosphodiesterase-5 inhibition mimics intermittent reoxygenation and improves cardioprotection in the hypoxic myocardium. PLoS One. 2011;6(11):e27910. 22140481 10.1371/journal.pone.0027910 PMC3225385

[38] Lepore JJ Maroo A Pereira NL , et al. Effect of sildenafil on the acute pulmonary vasodilator response to inhaled nitric oxide in adults with primary pulmonary hypertension. Am J Cardiol. 2002;90(6):677–680. 12231108 10.1016/s0002-9149(02)02586-9

[bibr39-10742484211014162] Schermuly RT Kreisselmeier KP Ghofrani HA , et al. Chronic sildenafil treatment inhibits monocrotaline-induced pulmonary hypertension in rats. Am J Respir Crit Care Med. 2004;169(1):39–45. 12958054 10.1164/rccm.200302-282OC

[bibr40-10742484211014162] Lepore JJ Maroo A Bigatello LM , et al. Hemodynamic effects of sildenafil in patients with congestive heart failure and pulmonary hypertension: combined administration with inhaled nitric oxide. Chest. 2005;127(5):1647–1653. 15888841 10.1378/chest.127.5.1647

[bibr41-10742484211014162] Zhao L Mason NA Morrell NW , et al. Sildenafil inhibits hypoxia-induced pulmonary hypertension. Circulation. 2001;104(4):424–428. 11468204 10.1161/hc2901.093117

[bibr42-10742484211014162] Lyons JM Duffy JY Wagner CJ Pearl JM . Sildenafil citrate alleviates pulmonary hypertension after hypoxia and reoxygenation with cardiopulmonary bypass. J Am Coll Surg. 2004;199(4):607–614. 15454147 10.1016/j.jamcollsurg.2004.06.003

[43] Ghofrani HA Osterloh IH Grimminger F . Sildenafil: from angina to erectile dysfunction to pulmonary hypertension and beyond. Nat Rev Drug Discov. 2006;5(8):689–702. 16883306 10.1038/nrd2030 PMC7097805

[44] Palloshi A Fragasso G Piatti P , et al. Effect of oral L-arginine on blood pressure and symptoms and endothelial function in patients with systemic hypertension, positive exercise tests, and normal coronary arteries. Am J Cardiol. 2004;93(7):933–935. 15050504 10.1016/j.amjcard.2003.12.040

[45] Nagaya N Uematsu M Oya H , et al. Short-term oral administration of L-arginine improves hemodynamics and exercise capacity in patients with precapillary pulmonary hypertension. Am J Respir Crit Care Med. 2001;163(4):887–891. 11282761 10.1164/ajrccm.163.4.2007116

[46] Brown MB Kempf A Collins CM , et al. A prescribed walking regimen plus arginine supplementation improves function and quality of life for patients with pulmonary arterial hypertension: a pilot study. Pulm Circ. 2018;8(1):2045893217743966. 29199900 10.1177/2045893217743966 PMC5731727

[47] Barbato E Herman A Benit E , et al. Long-term effect of molsidomine, a direct nitric oxide donor, as an add-on treatment, on endothelial dysfunction in patients with stable angina pectoris undergoing percutaneous coronary intervention: results of the MEDCOR trial. Atherosclerosis. 2015;240(2):351–354. 25875387 10.1016/j.atherosclerosis.2015.03.045

[48] Andersen CU Mulvany MJ Simonsen U . Lack of synergistic effect of molsidomine and sildenafil on development of pulmonary hypertension in chronic hypoxic rats. Eur J Pharmacol. 2005;510(1-2):87–96. 15740728 10.1016/j.ejphar.2005.01.020

[49] Jackson G Benjamin N Jackson N Allen MJ . Effects of sildenafil citrate on human hemodynamics. Am J Cardiol. 1999;83(5A):13C–20C. 10.1016/s0002-9149(99)00043-0 10078538

[50] Boolell M Allen MJ Ballard SA , et al. Sildenafil: an orally active type 5 cyclic GMP-specific phosphodiesterase inhibitor for the treatment of penile erectile dysfunction. Int J Impot Res. 1996;8(2):47–52. 8858389

[51] Beghetti M Gorenflo M Ivy DD Moledina S Bonnet D . Treatment of pediatric pulmonary arterial hypertension: a focus on the NO-sGC-cGMP pathway. Pediatr Pulmonol. 2019;54(10):1516–1526. 31313530 10.1002/ppul.24442 PMC6771736

[52] Nakamura T Zhu G Ranek MJ , et al. Prevention of PKG-1alpha oxidation suppresses antihypertrophic/antifibrotic effects from PDE5 inhibition but not sGC stimulation. Circ Heart Fail. 2018;11(3):e004740. 29545395 10.1161/CIRCHEARTFAILURE.117.004740 PMC5858464

[53] Salloum F Yin C Xi L Kukreja RC . Sildenafil induces delayed preconditioning through inducible nitric oxide synthase-dependent pathway in mouse heart. Circ Res. 2003;92(6):595–597. 12637371 10.1161/01.RES.0000066853.09821.98

[54] Marechal G Gailly P . Effects of nitric oxide on the contraction of skeletal muscle. Cell Mol Life Sci. 1999;55(8-9):1088–1102. 10442090 10.1007/s000180050359 PMC11147106

[55] Reid MB . Role of nitric oxide in skeletal muscle: synthesis, distribution and functional importance. Acta Physiol Scand. 1998;162(3):401–409. 9578386 10.1046/j.1365-201X.1998.0303f.x

[56] Worthley MI Horowitz JD Zeitz CJ . Lack of inotropic effect of nitric oxide on the rat myocardium. Clin Exp Pharmacol Physiol. 2005;32(7):526–530. 16026510 10.1111/j.1440-1681.2005.04225.x

[57] Hunter CJ Dejam A Blood AB , et al. Inhaled nebulized nitrite is a hypoxia-sensitive NO-dependent selective pulmonary vasodilator. Nat Med. 2004;10(10):1122–1127. 15361865 10.1038/nm1109

[58] Munzel T Daiber A . Inorganic nitrite and nitrate in cardiovascular therapy: a better alternative to organic nitrates as nitric oxide donors? Vascul Pharmacol. 2018;102:1–10. 29174923 10.1016/j.vph.2017.11.003

[59] Jankov RP Daniel KL Iny S , et al. Sodium nitrite augments lung S-nitrosylation and reverses chronic hypoxic pulmonary hypertension in juvenile rats. Am J Physiol Lung Cell Mol Physiol. 2018;315(5):L742–L751. 30091380 10.1152/ajplung.00184.2018

[60] Mondejar-Parreno G Moral-Sanz J Barreira B , et al. Activation of Kv 7 channels as a novel mechanism for NO/cGMP-induced pulmonary vasodilation. Br J Pharmacol. 2019;176(13):2131–2145. 30883701 10.1111/bph.14662 PMC6555858

[61] Barnes H Brown Z Burns A Williams T . Phosphodiesterase 5 inhibitors for pulmonary hypertension. Cochrane Database Syst Rev. 2019;1(1):Cd012621. 30701543 10.1002/14651858.CD012621.pub2 PMC6354064

